# Color changes and shear bond strength to simulated caries lesions treated with a novel solution of 20% silver nanoclusters in polymethacrylic acid

**DOI:** 10.1038/s41598-022-19757-6

**Published:** 2022-09-14

**Authors:** Gustavo Fabián Molina, María Belén Cabalén, Juan Pablo Aranguren, Santiago Daniel Palma, Gustavo Ariel Pino, Michael F. Burrow

**Affiliations:** 1 Facultad de Ciencias de la Salud, Universidad Católica de Córdoba, Córdoba, Argentina; 2 INFIQC: Instituto de Investigaciones en Fisicoquímica de Córdoba (CONICET-UNC), Córdoba, Argentina; 3 Departamento de Fisicoquímica, Facultad de Ciencias Químicas-Universidad Nacional de Córdoba, Córdoba, Argentina; 4 Centro Láser de Ciencias Moleculares-Universidad Nacional de Córdoba, Córdoba, Argentina; 5 Division of Restorative Dental Sciences, Prince Philip Dental Hospital, 34 Hospital Road, Sai Ying Pun, Hong Kong, China; 6 Unidad de Investigación y Desarrollo en Tecnología Farmacéutica (UNITEFA), CONICET, Córdoba, Argentina; 7 Departamento de Ciencias Farmacéuticas, Facultad de Ciencias Químicas, Universidad Nacional de Córdoba, Córdoba, Argentina

**Keywords:** Nanoscale materials, Bonded restorations

## Abstract

The aims of the study were: (1) To compare the staining effect on demineralized dentin simulating caries between silver nanoclusters (AgNCls) synthesized using polymethacrylic acid (PMAA) and silver diammine fluoride (SDF), and (2) to measure the shear bond strength (SBS) of a glass ionomer cement (GIC) to simulated caries lesions with and without the application of AgNCls/PMAA or SDF. Dentine blocks 4 mm thick from twenty-four non-carious third molars were sectioned and coated with nail varnish (Revlon, New York, USA). Simulated caries lesions on occlusal dentin surfaces were created (66 h in 0.05 M acetate buffer 2.2 mM calcium/phosphate pH 5.0). Specimens were divided into groups and treated with (n = 8): (A) 20% AgNCls/PMAA; (B) SDF 38% (Fagamin, Tedequim, Córdoba, Argentina); or (C) without treatment. AgNCls/PMAA or SDF were applied on the exposed surfaces with a microbrush for 10 s. Samples were incubated for 24 h at 37 °C at 100% relative humidity. Surface color was measured according to the CIE-L*a*b* system before and after demineralization (R0 and R1), 24 h and one week after treatment (R2 and R3), using a spectrophotometer (CM-600D Konica Minolta Sesing Inc., Japan). Groups A and B received an extra application of AgNCls/PMAA or SDF before a conventional GIC (Fuji IX-Gold Label, GC Corp, Tokyo, Japan) was bonded using a mold, 4 mm diameter × 3 mm high. For SBS, a Universal Testing Machine (Digimess RS-8000-5, China)—crosshead speed of 1 mm/min—was used. Statistical analysis was performed using ANOVA, Student-*t* and Scheffe-test at a significance of p < 0.05. Group A presented a stable color p = 0.24 between R1-R2 and R1-R3 in contrast to significant color changes in Group B (p = 0.02). SBS was higher (p < 0.01) in Group A (10.4 ± 2.7 MPa) compared to Groups B (3.3 ± 1.3 MPa) and C (4.0 ± 0.4 MPa), where no differences between the latter groups were observed (p = 0.77). Results of this preliminary study demonstrated that 20% AgNCls/PMAA did not stain simulated carious dentin and improved SBS of the GIC. The relevance of this study relies on the development of a therapeutic system to potentially arrest caries lesions without staining.

## Introduction

Silver Diammine Fluoride (SDF) is increasingly gaining more interest as an effective nonrestorative caries treatment alternative^1–9^. Increase of surface hardness and its antibacterial effects on cariogenic biofilm have been the highlights of this material, especially using protocols with a concentration of 38%^2,3,6,7^. However, staining of tooth structures caused by oxidation of silver ions is still considered a significant drawback of this cariostatic agent^10,11^. Innovative technologies have been developed to overcome these negative effects, for example, reducing silver ions down to nanoscale sizes to successfully avoid the oxidative staining^12^. Silver nanoparticles (AgNPs) have been used in dental materials for caries management therapies eg., in adhesive systems, toothpastes, and restorative materials. AgNPs can inhibit the growth and adhesion of cariogenic bacteria, especially *Streptococcus mutans* (*S. mutans*)^13,14^.

In cavitated lesions where cariogenic biofilm accumulates thus favoring the progression of caries, the use of restorative materials is advised to restore the defects and smoothen the surface that will allow effective cleansing^9^. Glass ionomer cements (GICs) are commonly used alone or in combination with SDF, both to arrest the progression of the lesion and to restore tooth anatomy^9,15^. Considering that the optimal seal of the cavity would enhance the ability to arrest the lesion and ensure longevity of the restoration, improving the adhesion of the GIC to demineralized dentin should promote a better performance and increase restoration and tooth longevity. However, no beneficial effect has been observed when a GIC is bonded to SDF-treated surfaces in comparison to the traditional 10% polyacrylic acid conditioning that has proven to increase the shear bond strength values to dentin^16,17^.

The present study tested a novel product which is expected to combine the antibacterial properties of AgNPs as well as the potential to promote adhesion to dentin by means of using a polycarboxylic acid as a liquid precursor. Therefore, the aims of this study were: (1) To compare the staining effect on demineralized (simulated caries) dentin between silver nanoclusters (AgNCls) synthesized using polymethacrylic acid (PMAA) and silver diammine fluoride (SDF), and (2) to measure the shear bond strength (SBS) of a conventional glass ionomer cement (GIC) to simulated carious lesions with and without the application of AgNCls/PMAA or SDF.

## Materials and methods

All experimental protocols were approved by the secretary of research and development, Universidad Católica de Cordoba, Argentina (SI-UCC research grants) and by the National Agency for Research under the research grant FONCYT-PICT2020 Serie A #00539, and PICT2019 N° 241, CONICET-PIP, PRIMAR2017 (SeCyT-UNC).

All methods were carried out in accordance with relevant guidelines and regulations.

### Development and characterization of the Silver nanocluster experimental agent

Different polymers derived from carboxylic acids, such as polyacrylic acid (PAA), polymethacrylic acid (PMAA) and polymethyl vinyl ether-alt-maleic anhydride (pMVEMA) have been used as liquid precursors for the transport and stabilization of AgNCls^18–24^. In the present development, the synthesis of h-AgNCls was carried out at room temperature by photoreduction of AgNO_3_ in the presence of PMAA with 355 nm/wavelength light, according to what has been reported in the literature^18–24^. In all cases, the optimal pH conditions were constantly evaluated, in the range pH 5.5–6.5.

The solutions obtained were characterized by fluorescence and absorption spectroscopy, and the particle size was determined by dynamic light scattering (DLS) and AFM microscopy.

The concentration of AgNO_3_ was 5 × 10^–4^ M with a 5:1 Ag:monomer ratio in the initial mixture solution of Ag^+^/PMAA, which has been shown to have the best antibacterial properties^21^. These developmental procedures have been previously reported using a different platform and with a resin-based polymeric liquid precursor^25^.

### Sample size

A two-tailed test was used to determine the sample size using the proportional comparison formula considering 5% for the significance level and 80% for the statistical power. For that purpose, the results obtained in a study that reported SBS values of GICs to dentin surface treated with SDF were considered for reference^16^, resulting in ≥ 7 samples needed for each group.

### Preparation of samples

Twenty-four non-carious third molars were obtained from the Bank of Human Teeth, Faculty of Dentistry, Universidad Nacional de Córdoba, Argentina (Ord. 3/16 HCD and Res. 333/17 HCD). Teeth were sterilized via gamma irradiation for 24 h before sectioning. Dentine blocks, 4 mm thick, were obtained by removing the occlusal enamel using a water-cooled low-speed cutting machine (Buehler, Germany) perpendicular to the long axis of the tooth to obtain flat mid-coronal dentin surfaces. These were subsequently polished with 400-grit silicon carbide paper and coated with nail varnish (Revlon, New York, USA), exposing a 5 × 5 mm window on the occlusal dentin surface for production of demineralized dentin to simulate dental caries.

Samples were immersed for 66 h in a solution containing 0.05 M acetate buffer, 2.2 mM calcium phosphate adjusted to pH 5.0 to generate a demineralized layer approximately 150-μm deep to simulate a carious lesion.

Once the artificial lesions were produced, specimens were divided in two treatment groups (A and B) and a control group (C) without surface treatment (n = 8):

(A) treated with 20% AgNCls/PMAA; the solution was applied onto the exposed demineralized surface with a microbrush for 10 s then incubated at 37 °C and 100% relative humidity for 24 h.
(B) treated with SDF 38% (Fagamin, Tedequim, Córdoba, Argentina); the solution was applied on the exposed demineralized surface with a microbrush for 10 s then incubated at 37 °C and 100% relative humidity for 24 h.
(C) Control (no treatment); exposed demineralized surfaces were left untreated then incubated for 24 h at 37 °C and 100% relative humidity.

After 24 h incubation, samples were tested for initial color changes after the application of the different treatments and immersed again for a further 6 days at 37 °C and 100% relative humidity. Final readings to determine color variations were done after 7 days after having received a single application of the respective treatments. The shear bond strength (SBS) of a high-viscosity glass ionomer was tested after the evaluation of color variations was finalized.

### Color changes

Color measurements were obtained using a spectrophotometer (CM-600D Konica Minolta Sesing INC, Japan) and all measurements were replicated three times from which a mean value was calculated and considered as the final value. Before color testing, the spectrophotometer was calibrated using the specified calibration plate. The CIE-L*a*b* color system, which is defined as a 3-dimensional (3D) measurement system, was applied to interpret the readings: ‘L’ indicates the brightness, ‘a’ red-green, and ‘b’ the yellow-blue proportion of the color^26^. The obtained values were automatically stored digitally by a computer connected to the spectrophotometer. Specific color coordinate differences (ΔL, Δa, Δb) were recorded before and after demineralization of the samples (R0 and R1), 24 h (R2) and one week after application of the respective treatment (R3).

Total color differences (ΔE) were calculated using the following formula: ΔE = ((ΔL)^2^ + (Δa)^2^ + (Δb)^2^)^1/2^.

For assessing the influence of the treatment options on color change to demineralized dentin, Groups A and B were compared to a control group, where demineralized dentin was left untreated (Group C).

### Shear bond strength test

Groups A and B received a second application of AgNCls/PMAA or SDF prior to placement of a GIC—instead of a conditioner—to potentially enhance the adhesion of the GIC. A high-viscosity conventional glass ionomer cement (Fuji IX-Gold Label, GC Corp, Tokyo, Japan) was hand-mixed on a pad for 20–30 s, following the manufacturer’s instructions, and then inserted with a plastic spatula into a mold 4 mm diameter × 3 mm high which was positioned on demineralized treated or untreated (control) dentin surfaces. A glass slab coated with Vaseline® was placed on top of the molds during the setting of the GIC, and held together with a clamp for 5 min. Following the initial set, specimens were then stored in 100% relative humidity at 37 °C for 24 h prior to bond testing.

For SBS, specimens were placed in a jig attached to a Universal Testing Machine (Digimess RS-8000-5, China). Specimens were loaded using a beveled flat blade placed as close as possible to the bonded interface then stressed in shear at a crosshead speed of 1 mm/min until failure^27^. SBS values, expressed in MPa, were calculated using the following formula: (MPa) = N/12.6 where N is the force applied in Newtons at the moment of failure, divided by the bonded surface area of the sample.

The failure mode of each specimen was analyzed using a confocal laser scanning microscope (OLYMPUS LEXT OLS4000, Tokyo, Japan) at a low magnification (100X). Failure was determined as one of three possible modes, namely, adhesive, cohesive or mixed failure.

Statistical analysis was performed using ANOVA, Student-*t* and post hoc Scheffe-test with a significance set at the 95% confidence level (p < 0.05).

**Figure 1:**
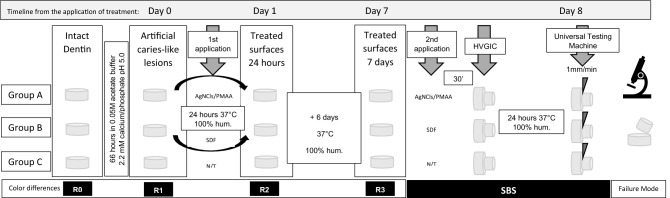
Timeline of the study. Sequence for testing color changes and SBS of the samples.

Figure 1 shows the timeline for the study and the sequence applied to groups for their comparison.

### Ethics approval and consent to participate

Teeth used in the study were obtained from the Bank of Human Teeth, Faculty of Dentistry, Universidad Nacional de Córdoba, Argentina (Ord. 3/16 HCD and Res. 333/17 HCD) and consent for their use was waived according to ethics regulations.

## Results

### Color changes

Table 1 shows the color differences of each treatment group between each of the consecutive readings. R0 was performed on intact dentin whereas R1 measured color coordinates of demineralized dentin. As this study aimed to compare the effect on color stability of two treatment options applied on demineralized dentin, R1 was considered the baseline reading for comparisons. No significant color variations were detected after demineralization of the dentin surface (R0–R1) although changes were more visible among groups from the moment the specimens were subjected to the different treatments.

**Table 1:** Mean color differences and standard deviations of treatment groups between each reading.

Treatment group	n	∆E O–1	∆E 1–2	∆E 2–3
Group A	8	**8.5** ± 5.8	**4.4** ± 2.9	**4.3** ± 2.2
Group B	8	**9.1** ± 4.1	**8.3** ± 2.6	**9.4** ± 4.9
Group C	8	**8.7** ± 5.4	**2.2** ± 1.3	**3.6** ± 4.5

Ref. **∆E: Color difference—**No significant differences were found between the three groups and within each group, between readings.

The effect of the surface treatment is shown in Table 2, where it was noted that Group A presented color stability (p = 0.24) from demineralized dentin and after 24 h and 7 days of applying the AgNCls/PMAA solution (R1-R2 and R1-R3). In contrast, significant color changes were observed in Group B (p = 0.02) after SDF application to the demineralized dentin surfaces.

**Table 2:** Mean color differences and standard deviations of treatment groups between the application of the surface treatment and the subsequent readings.

Treatment option	N	∆E 1–2	∆E 1–3	*p* (t-Test)
Group A	8	**4.4** ± 2.9	**2.9** ± 1.7	0.24
Group B	8	**8.3** ± 2.6	**12.6** ± 3.8	0.02*
Group C	8	**2.2** ± 1.3	**2.7** ± 1.6	0.68

Ref. ∆E: Color difference—*significant differences.

### Shear Bond Strength test

As displayed in Table 3, SBS values were higher (p < 0.01) after treatment with AgNCls/PMAA (Group A) compared to SDF (Group B) and the control group (Group C) (p < 0.05). No significant differences were found between the two latter two groups (p > 0.05).

**Table 3:** Shear Bond Strength mean values and standard deviations of the three groups.

Treatment group	N	SBS (MPa)	ANOVA (*p-value*)
Group A	8	**10.4** ± 2.7^a^	5.7E−08
Group B	8	**3.3** ± 1.3^b^	
Group C	8	**4.0** ± 0.4^b^	

Ref. SBS Values expressed in megapascal (MPa); Different letters express statistical differences ^a,b^p < 0.05 (Scheffé test).

All samples that received no pre-treatment or treated with SDF showed adhesive failures whereas three of the eight specimens that were pre-treated with AgNCls/PMAA showed remnants of GIC in combination with areas of adhesive failure on the surface (mixed failure mode).

## Discussion

The present study represents a twist on a preliminary attempt for the development of a non-restorative caries infiltration technology. In a previous experimental design, the polymeric precursor that was used had been a resin-based liquid that served as a carrier for zinc-oxide nanoparticles (ZnNPs)^25,28^. A laboratory study showed that, although a significant increase of surface hardness occurred after infiltration into demineralized dentin, no traces of ZnNPs were found to penetrate greater than 20 µm into simulated dentin caries lesions^28^. The uncertainty of the penetration depth that such a polymer achieved into the demineralized dentin surface became a major difficulty to overcome. Therefore, a new proposal introduced AgNCls instead of ZnNPs that could be tracked by means of fluorescence and was also able to be synthesized in a lower viscosity liquid precursor. In addition, the polymethacrylic acid (PMAA) was also associated with chemical interactions with the collagen fibers in the demineralized dentin.

However, the use of silver ions is associated with one of the major weaknesses of SDF, which is the darkening of treated carious lesions^29^. A laboratory study concluded that staining of carious tissue can be avoided by using nano-sized silver particles protected with a capping agent that may prevent oxidative formation of silver chloride^12^. That study combined AgNPs coated with polyethylene glycol (AgNPs-PEG) and sodium fluoride (NaF) to arrest caries by means of antibacterial and remineralizing properties of its compounds without staining the treated surfaces.

The color difference value (ΔE*) resulting from solutions containing AgNPs-PEG and NaF ranged from 2.8 to 8.5 whereas the discoloration due to SDF (ΔE* = 81.6) was more than 10 times higher than the experimental product^12^. These results are similar to those obtained in the present study, although the color difference was not that great for the SDF treatment group. Nevertheless, there is a common outcome observed in both studies with a lack of staining when the silver particles are reduced to nanoparticles or nanoclusters.

In the previously mentioned study, AgNPs were protected by polyethylene glycol (PEG) to avoid oxidative contamination, thus less staining side effects were expected, whereas in the present study, the protective function relied on the polymethacrylic acid (PMAA).

Potassium iodide (KI) has proved to reduce discoloration caused by SDF, however its effect on the SBS of GICs to caries-affected dentin was found to be neither beneficial nor detrimental in previous laboratory studies^30^. In the present study, significant differences were found in bond strength of GICs to simulated caries lesions using AgNCls/PMAA compared to SDF or without treatment of the surface. This improvement was also observed in the failure mode of the samples, with remnants of the GIC on the dentin surface after failure in the specimens that had received the application of AgNCls/PMAA.

After the final reading to assess color changes was completed, Groups A and B received a second application of AgNCls/PMAA or SDF prior to placement of a GIC. The purpose of this second application was to test the effect of each treatment as a conditioner to potentially enhance the adhesion of the GIC. Although the samples had already received one respective treatment application, the following seven days of incubation may have altered the chemical activation of radicals on the surface of the artificial caries lesions, hence the second application.

It is hypothesized that PMAA may have played a fundamental role not only to improve the bonding of the glass ionomer cement (GIC) but also achieved some intrafibrillar infiltration into the collagen fiber network of the demineralized dentin. The theory of using a polymer induced liquid precursor (PILP) as a vehicle for transporting ions into the collagen fiber network has been proven in laboratory studies to be an effective source to reverse the demineralization process caused by caries activity in tooth structures^31–33^. PMAA belongs to the group of polyanionic acids which show similar chemical reactions as polyacrylic acid (used as a cavity conditioner to enhance bonding of GIC to dentin) and to polyaspartic acid (PASP) that is the liquid precursor described for functional mineralization^31^.

Nevertheless, the assumption of the relevance of including AgNCls in PMAA should be further tested with the aid of advanced analytical techniques, such as cryogenic transmission electron microscopy (cryo-TEM), in situ atomic force microscopy (AFM)^34^, nuclear magnetic resonance (NMR), and modeling simulations to determine whether this mechanism occurs with PMAA as demonstrated in the PILP theory.

Based on the evidence of a positive effect on bond strength of GICs to dentin after pretreatment of 10% polyacrylic acid (PAA), the authors had considered the synthesis AgNCls using this polymeric acid. However, photoreduction was not possible to achieve an outcome as effectively as that with PMAA. Although further evaluation is needed to determine whether PMAA is as biologically acceptable as PAA, the results of this study confirmed that it has a positive effect on the shear bond strength of GICs to demineralized dentin.

From a clinical perspective, the use of SDF (non-restorative treatment option) instead of a restorative treatment option such as the ART approach or even a conventional approach is still a matter of debate, depending on caries risk assessment^35^. The decision to remove or to remineralize caries-affected structures has been set for cavitated lesions using selective removal until firm dentin (caries-affected dentin that can be remineralised) is reached according to a recent consensus of experts^35^. However, the controversy of relying on ultraconservative strategies or arresting lesions without any surgical procedure remains as the progression of the lesions might not be completely arrested and further demineralization might occur in the deeper parts of the lesion. Therefore, the combination of these latter options has been suggested as a potential synergy, both arresting the lesion and sealing the cavity with a fluoride-releasing material, known as the SMART technique (Silver Modified Atraumatic Restorative Treatment)^15,36^.

The development of such an agent based on AgNCls/PMAA focuses on enhancing this approach by using the beneficial antibacterial features of silver nanoparticles while avoiding the staining, that remains an esthetic problem even beneath a restorative GIC, as well as by improving the bonding ability of GIC to the treated surface.

The results obtained in this study feature only some of the properties that characterize a potentially effective product for arresting caries lesions while enhancing the adhesion of filling materials to restore tooth structure. These results should be added to biological tests to assess cytotoxicity and antibacterial properties as well as the ability to recover the physico-mechanical characteristics of the dentin to compete with the caries-arresting effects of SDF^12,29^. Nevertheless, despite the limitations, the current outcomes encourage the pursuit of further studies on the use of AgNCls since it appears to have eliminated two of the main disadvantages of SDF: staining and bond strength of restorative cements to the treated surface.

## Conclusion

Results of this preliminary study demonstrated that the use of a 20% AgNCls/PMAA solution to treat simulated caries lesions did not stain the demineralised dentin and improved the adhesive strength of a conventional glass ionomer cement to the treated surface in comparison to a 38% SDF solution.

## Data Availability

The datasets generated and/or analysed during the current study are not publicly available due to confidentiality reasons of a novel product that is being under development, but are available from the corresponding author upon reasonable request.
